# Assessment of chemical-shift and diffusion-weighted magnetic resonance imaging in differentiating malignant and benign vertebral lesions in oncologic patients. A single institution experience

**DOI:** 10.2478/raon-2024-0049

**Published:** 2024-10-04

**Authors:** Marija B Mijaljevic, Zorica C Milosevic, Slobodan Đ Lavrnic, Zorica M Jokovic, Danica I Ninkovic, Radoje M Tubic, Rajna R Jankovic

**Affiliations:** Department of Radiology, Institute of Oncology and Radiology of Serbia, Belgrade, Serbia; Faculty of Medicine, University of Belgrade, Belgrade, Serbia; Department of Radiology, University Children's Hospital, Belgrade, Serbia

**Keywords:** magnetic resonance, chemical-shift imaging, diffusion-weighted imaging, bone marrow lesions

## Abstract

**Background:**

To analyze the contribution of two non-standard magnetic resonance imaging (MRI) techniques the chemical-shift image (CSI), and diffusion-weighted imaging (DWI) in distinguishing malignant and benign vertebral bone marrow lesions (VBMLs).

**Patients and methods:**

Conventional spine MRI protocol, followed by CSI and DWI was performed with a 1.5 T system on 102 oncologic patients between January 2020 and December 2023. From the identified 325 VBMLs, 102 representative lesions (one per patient) were selected. VBMLs were divided into malignant (n = 74) and benign (n = 28) based on histopathology, or imaging follow-up. The quantitative parameters for VBMLs assessment were signal intensity ratio (SIR) derived from CSI and apparent diffusion coefficient (ADC) derived from DWI.

**Results:**

The malignant VBMLs had significantly higher SIR values (p < 0.05) and lower ADC values compared to benign VBMLs (p < 0.05). The area under the curve (AUC) was 0.953 (p < 0.001) for SIR, and 0.894 for ADC (p < 0.001) (cut-off at > 0.82, and ≤ 1.57x10^−3^ mm^2^/s, respectively). The sensitivity and specificity for SIR were 93.6%, and 88.5%, while for ADC were 88.2% and 92.3% (respectively). The combined use of SIR and ADC improved the diagnostic accuracy to AUC of 0.988 (p < 0.001, cut-off at > 0.19), sensitivity, and specificity of 100.0% and 90.9% (respectively).

**Conclusions:**

Quantitative parameters, SIR and ADC, derived from two non-standard MRI techniques, CSI, and DWI, showed diagnostic strength in differentiating malignant and benign VBMLs. Combining both methods can further enhance the diagnostic performance and accuracy of spine MRI in clinical practice.

## Introduction

Differentiating benign from malignant lesions in the spinal column is one of the key goals of neurooncological imaging. Skeletal metastases are the most common malignant tumors of the bone system in adults, with a high incidence, especially in the case of breast and prostate cancer, where they account for up to 70% of cases.^1^ The spinal column is a frequent site of metastases, involving various structures such as bone, epidural space, leptomeninges, and spinal cord. Metastases of the spinal column tend to be multiple. For instance, in breast cancer, they are commonly found in the thoracic and lumbar regions, followed by the cervical region (63.6%, 53.8%, and 21.7%, respectively).^2^ MRI of the spinal column has a high sensitivity and specificity in detecting metastases in bone structures (91%, and 95%, respectively).^1,3^ However, on a conventional MRI examination of the spinal column, malignant and some benign lesions appear identical, representing a radiological diagnostic challenge. The conventional MRI examination sometimes can not differentiate benign from malignant fractures.^4^ Furthermore, a personal history of cancer does not necessarily imply malignant vertebral body infiltration and benign spine lesions in oncologic patients can cause diagnostic dilemmas, such as incidental findings of preformed benign lesions, or osteoporotic vertebral fractures due to endocrine therapy for breast cancer.^5^

Clinical work-up pathways of malignant and benign vertebral bone marrow lesions (VBMLs) are divergent, and differentiation of etiology is crucial. The development and application of non-standard MRI techniques for spine examination are aimed at increasing the diagnostic accuracy of MRI examination. One such technique is a gradient-echo MRI technique of chemical-shift imaging (CSI) in-phase (IP) and out-of-phase (OOP). The physicochemical principle of the CSI IP-OOP MRI technique is based on the different oscillation frequencies of water and fat protons.^6^ Due to the presence of water and fat in normal fatty or hematopoietic red bone marrow, on the CSI-OOP sequence signal intensity (SI) of the bone marrow fat is suppressed. On the contrary, the complete replacement of normal bone marrow by malignant cells should result in lack of fat suppression on the oposite phase images.^7^

Diffusion-weighted magnetic resonance imaging (DWI) is another non-standard MRI technique for differentiating VBMLs. Quantifying the diffusion of water molecules in tissue through a numerical parameter – the apparent diffusion coefficient (ADC)- shows that the ADC value of normal bone marrow is 0.2–0.6 x 10^−3^ mm^2^/s.^8^ This represents a physiological restriction of water diffusion due to the anatomical trabeculated structure of the vertebral spongiosa and fat in the bone marrow. The disrupted bone structure in pathologically altered bone marrow leads to different ADC values, depending on the type of pathology. However, the degree of increase of ADC value in benign lesions is higher compared to malignant lesions.^9^

In the era of a transition to artificial intelligence and machine learning in neuroimaging, the spine MRI examination with different imaging sequences and optimization of protocols still represent an important part of the clinical routine in oncologic institutions. Our study aimed to analyze and combine two non-standard MRI techniques, CSI IP-OOP, and DWI, as additional methods to conventional spine MRI in distinguishing malignant and benign VBMLs.

## Patients and methods

The retrospective study was conducted at the Institute of Oncology and Radiology of Serbia. The study was in accordance with the ethical standards of the institutional and national research committee and was approved by the Ethics committee of our institution (No. 284-01/2022/3). Each patient completed and signed an informed consent for the MRI examination.

### Patients characteristics

The spine MRI examinations performed between January 2020 and December 2023 were reviewed. The inclusion criteria were as follows: (1) pathohistologically verified primary malignancy; (2) initial conventional spine MRI examination to determine the clinical stage of the disease; (3) follow-up conventional MRI examination after completed oncological treatment, including the patients with and without clinical suspicion of metastases in the spinal column; (4) CSI IP-OOP and DWI techniques; (5) applied one or more of the following procedures: biopsy with pathohistological (PH) verification of VBML; scintigraphy (Sci); positron emission tomography/computed tomography (PET/CT); adequate clinical and neuroradiological follow-up for 6 months or longer after the detection of lesion(s), which will ensure the final etiology. Apart from general contraindications to MRI, the exclusion criteria were as follows: (1) a focal lesion < 1 cm in diameter; (2) systemic anticancer therapy and/ or radiotherapy in the region of interest completed 6 months before MRI examination; (3) inability of the patient to adequately withstand the examination; (4) inappropriate MRI views of the spinal column for technical reasons; (5) patients under 18 years of age.

According to the mentioned criteria, 102 successive patients with 325 VBMLs were enrolled in the study, 85 women (83%) and 17 men (17%). The mean age of patients was 61.8 years (range 30–85 years). The malignancies were: breast cancer in 62 patients (60.8%), lung cancer in 10 (9.8%), prostate cancer in 7 (6.9%), melanoma in 4 (3.9%), and others – a total of 19 patients (18.6%), i.e ≤ 2 patients with one of the following histological types: colorectal cancer, cervical cancer, endometrial cancer, renal cell carcinoma, oesophageal cancer, nasopharyngeal cancer, laryngeal cancer, hepatocellular carcinoma, salivary gland cancer, multiple myeloma, lymphoma, medulloblastoma, and malignant hemangioendothelioma.

The distribution of VBMLs in 102 patients was: thoracic spine in 51 (50.0%), lumbar spine in 38 (37.2%), sacrum in 12 (11.8%), and cervical spine in one patient (1.0%). Solitary VBML was found in 30 patients (29.4%) and multiple lesions in 72 patients (70.6%).

The VBMLs were classified as either benign or malignant. The diagnosis was made based on the biopsy and PH confirmation in 10 patients (9.8%). In the absence of HP confirmation, the diagnosis was made based on the additional imaging studies – Sci in 28 patients (27.5%) and PET/CT in 8 patients (7.8%), or the follow-up MRI examination. VMBLs that had MRI characteristics typical of benign lesions and had stable appearances during follow-up MRI examinations of at least 6 months were classified as benign.^10^

### MRI protocol

Data were acquired with a 1.5 T MRI system (Magnetom Avanto Fit, Siemens, Germany) using phased-array spine coils. The routine clinical MRI protocol used at our institution included T1-weighted (T1W), T2W, short tau inversion recovery (STIR) MRI sequences precontrast, T1W fat-suppressed (FS) with an intravenous bolus of a gadobutrol (Gd-DO3A-butrol; Gadovist, 0.1 mmol/ kg, 1 mmol/ml; Bayer Healthcare, Germany) contrast agent, at a rate of 3–5 ml/s. The parameters of routine clinical MRI protocol were: T1W TSE (TR/ TE 613/9.8), T2W TSE (TR/TE 3380/89), STIR TSE (TR/TE 2370/78; inversion time, 160 ms), T1WFS TSE after contrast medium application (TR/TE 881/9.8), slice thickness 3 mm (cervical), 4 mm (thoracic and lumbar spine), matrix size 384 x 384 mm.

The non-standard examination protocols were the CSI IP-OOP technique and DWI with ADC maps, performed in the sagittal plane. The image acquisition parameters of the CSI IP-OOP technique included FOV 262x350 mm, matrix size 426x320 mm, (TR/TE in phase 118/5.27, out of phase 118/2.35), thickness section 3 mm (cervical), 4 mm (thoracic and lumbar spine). DWI sequence was acquired before contrast agent administration with fat saturation single-shot TSE sequence (TR/TE 3000/99; 128 x 92 matrix, flip angle 180 degrees, using b values of 100 s/mm^2^ and 400 s/mm^2^).

### Image analysis

Images were analyzed using a Syngo via program (Siemens Medical Solutions, USA). The region of interest (ROI) was drawn manually and placed at a single slice with the largest possible lesion diameter, where the lesion was best seen on T1W, STIR, DWI, CSI-IP, and CSI-OOP sequences. Then it was copied to another image using the software paste option to the same image position. Areas close to the rim, lesions with intralesional hemorrhage, or necrotic areas were excluded from measurement. In the cases of numerous similar focal VBMLs in the same patient, ROI was chosen for only one of the most prominent lesions, to reduce the potential statistical influence of multiple similar lesions in single patients: 51 ROIs (50.0%) were located in the thoracal spine, 38 ROIs (37.3%) in the lumbar spine, 12 ROIs (11.7%) in the sacral and one ROI (1.0%) in the cervical spine.

On the CSI-IP and CSI-OOP sequences, SI of bone marrow was measured by manually placing the ROI. Signal intensity ratio (SIR) was calculated with the obtained SI values at ROI, according to the formula: “SIR = out-of-phase signal intensity value / in-phase signal intensity value” which was used in previous studies to distinguish benign from malignant bone marrow involvement.^11^

The software calculation of the ADC value was performed after manually placing the ROI on a representative part of the image. A methodology for defining the cut-off values of ADC between benign and malignant lesions was according to previous studies.^12^

Both quantitative image analyses, SIR, and ADC were performed in consensus by two radiologists with 10 years (M.M.) and 5 years (D.N.) of clinical experience in the field of MRI neuro-oncology and a physicist (S.G.) with more than 15 years of experience in CSI IP-OOP technique and DWI.^13^

All researchers were blinded to patient-related information, including histological types of lesions.

### Statistical analysis

The mean value, standard deviation, ranges and percentages were determined for parameters describing the study group. Receiver operating characteristic (ROC) and area under the curve (AUC) analyses, as well as the cut-off values, sensitivity, and specificity, were used to compare the diagnostic performance of the SIR, ADC, and combination (SIR, ADC) in terms of distinguishing focal benign VBMLs from metastases. Between-group differences in the SIR, and ADC values, were compared using the Mann-Whitney U-test. The multivariate regression analysis formula with SIR and ADC values was: y = −2,461–14,426*ADC + 28,085*SIR. Statistical analyses were performed using the software package R (version 4.2.3). For all assessments, a p-value < 0.05 was taken to indicate statistical significance.

## Results

**FIGURE 1.**
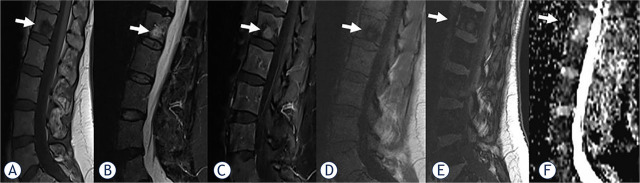
A 50-year-old woman with breast cancer and focal benign vertebral bone marrow lesion (VBML) (arrows). A round-shaped, abnormal signal intensity change in the bone marrow is evident in the L1 body on sagittal T1-weighted **(A)**, short tau inversion recovery (STIR), **(B)**, contrast-enhanced fat- satureted T1-weighted **(C)** in-phase T1-weighted **(D)**, out-of-phase T1-weighted **(E)** images. Apparent diffusion coefficient (ADC) **(F)** value is 1.74 x 10^−3^ mm^2^/s. The signal intensity ratio (SIR) value is calculated as 0.8 and is consistent with benignity. Standardized Uptake Value (SUV) on PET scan excluded malignancy. After one year of follow-up, the lesion STIR/postcontrast hyperintensity almost disappeared.

**FIGURE 2.**
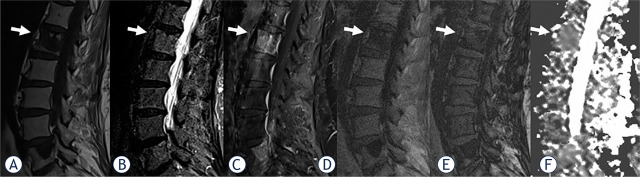
A 75-year-old woman with breast cancer and five focal malignant vertebral bone marrow lesions (VBMLs), the most prominent in the L1 body (arrows) which we chose to analyze. Abnormal signal intensity change is evident on sagittal T1-weighted **(A)**, short tau inversion recovery (STIR), **(B)**, contrast-enhanced fat-satureted T1-weighted **(C)** in-phase T1-weighted **(D)**, and out-of-phase T1-weighted **(E)**. Apparent diffusion coefficient (ADC) **(F)** value is 0.99 x 10^−3^ mm^2^/s. The signal intensity ratio (SIR) value is calculated as 1.07. which indicates the malignant lesion. Sci suggested malignant lesions.

Analysis of spinal MRI was performed in a total of 28 (27.5%) benign and 74 (72.5%) malignant VBMLs. The benign VBMLs were: vertebral fractures in 12 patients, atypical hemangiomas in 10 patients, Schmorl's nodes in three patients, focal hematopoietic islands in two patients, and aggressive vertebral body hemangioma in one patient. The malignant VBMLs were: metastases in 70 patients, spinal lymphomas in two patients, malignant hemangioendothelioma in one patient, and multiple myeloma in one patient. Representative images for benign and malignant VBMLs are given in Figure 1 and 2.

The median SIR value and range for the malignant lesions were 0.99 (0.78–1.37), while for the benign lesions, they were 0.65 (0.24–1.04). The median ADC value and range for the malignant lesions were 1.22 x 10^−3^ mm^2^/s (0.89–1.75 x 10^−3^ mm^2^/s), while for the benign lesions, they were 1.74 x 10^−3^ mm^2^/s (0.73–2.24 x 10^−3^ mm^2^/s). The SIR values of malignant lesions were significantly higher compared to benign lesions (p < 0.05), while the ADC values of malignant lesions were statistically significantly lower compared to benign lesions (p < 0.05).

**FIGURE 3.**
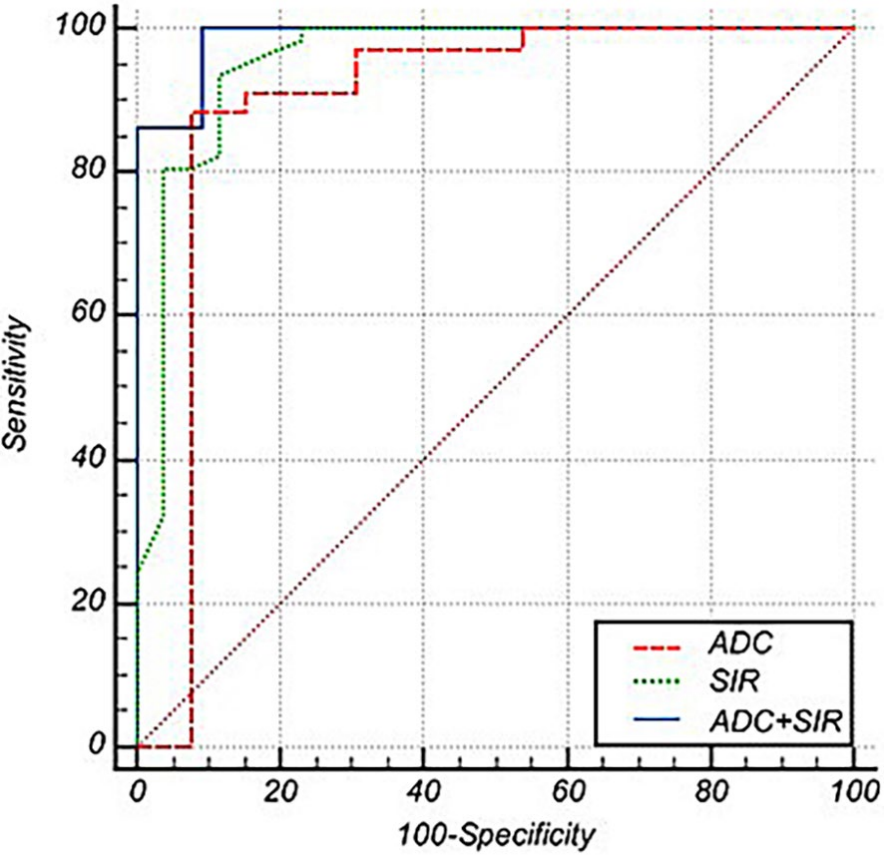
The receiver operating characteristic (ROC) curves of the apparent diffusion coefficient (ADC) (red line), signal intensity ratio (SIR) (green line), and combined SIR and ADC (blue line). The ROC curves show that the combined SIR and ADC have the highest AUC for differentiating benign from malignant vertebral bone marrow lesions (VBMLs), followed by the SIR and ADC.

The diagnostic performance of SIR, ADC, and their combination (SIR, ADC) in the differentiation of VBMLs is shown in Table 1 and Figure 3. The AUC, cut-off values, sensitivity, and specificity showed a high agreement of SIR and ADC in the differentiation of benign and malignant VBMLs. The combination of SIR and ADC demonstrated the best diagnostic performance (AUC = 0.988, 95% confidence interval [CI] = 0.872–1.000), with a sensitivity of 100.0% and specificity of 90.9%.

**TABLE 1.** Diagnostic performance of the signal intensity ratio (SIR), apparent diffusion coefficient (ADC), and combination (SIR, ADC) for differentiating benign from malignant vertebral bone marrow lesions (VBMLs)

**Parameters**	**AUC (95% CI)**	**Standard error**	**p**	**Cut-off**	**Sensitivity (%)**	**Specificity (%)**
SIR	0.953 (0.886–0.987)	0.029	< 0.001	> 0.82	93.6	88.5
ADC	0.894 (0.769–0.965)	0.077	< 0.001	≤ 1.57	88.2	92.3
Combination (SIR, ADC)	0.988 (0.872–1.000)	0.014	< 0.001	> 0.19	100.0	90.9

AUC = area under the curve; CI = confidence interval; p = significance level

Cut-off values are given in units of x10^−3^ mm^2^/s for ADC.

## Discussion

The number of patients with malignant tumors is increasing, and the medical research in the field of neuro-oncology represents a step closer to optimal therapy and potential cure for the patient.^14^ The development of bone metastases is a classical unfavorable prognostic factor, implying a palliative approach to the therapy and poor overall survival. However, technological advancements introduced various surgical procedures with curative intent for selected patients, particularly those with solitary bone metastases. Understanding different mechanisms related to the pathogenesis of bone metastases developed some promising systemic therapies targeting specific malignant cell types, advancing the concept of precision therapy in oncology.^15^ All these facts highlight the importance of imaging methods in guiding treatment decisions and improving patient outcomes.

In our study, out of a total of 102 VBMLs, 28 (27.5%) were classified as benign and 74 (72.5%) as malignant. Among the malignant lesions, metastases were the most common, accounting for 70 out of the 74 malignant lesions, while among the benign lesions, vertebral fractures (12 cases) and atypical hemangiomas (10 cases) were the most frequently observed lesions. It is worth mentioning that in the group of malignant VBMLs, extremely rare skeletal metastases of medulloblastoma, and malignant hemangioendothelioma were present.

The SIR values derived from the CSI IP-OOP technique in our study demonstrated high sensitivity (93.6%) and specificity (88.5%) in distinguishing between benign and malignant VBMLs. The AUC was 0.953 (95% CI, 0.886–0.987). Malignant VBMLs exhibited significantly higher SIR values than benign VBMLs, with a cut-off value of > 0.82. A comparison of our results with previous investigations supports the diagnostic performance of CSI. A study by Disler *et al*. in the late 1990s provided initial insights into the predictive capability of the SIR between in-phase and out-of-phase images for distinguishing neoplastic or non-neoplastic lesions, achieving a sensitivity of 100% and specificity of 94–100%.^16^ Similarly, studies by van Vucht *et al*. and Zajick *et al*. confirmed the accuracy of CSI in distinguishing between neoplastic and non-neoplastic lesions. However, Zajick *et al*. noted limitations in differentiating malignant bone tumors from non-fat-containing benign bone tumors.^17,18^ A meta-analysis by Suh *et al*. supported the efficacy of CSI in distinguishing between benign and malignant VBMLs, with high sensitivity, specificity, and an AUC of 0.95 (95% CI, 0.93–0.97), with sensitivity and specificity rates of 92% and 89%, respectively.^19^ Despite comparable results among the mentioned studies, variations existed in the histology characteristics of the lesions and the applied techniques (such as minimum TR and TE, flip angle, slice thickness, gradient-echo CSI, or the Dixon method). These differences may influence the overall diagnostic performance and should be considered when interpreting each study findings.

The ADC values derived from DWI in our study revealed notable sensitivity and specificity in distinguishing between benign and malignant VBMLs (88.2% and 92.3%, respectively). The AUC was 0.894 (95% CI, 0.769–0.965), indicating the strong discriminative ability of DWI. Malignant lesions were characterized by significantly lower ADC values (cut-off value of ≤ 1.57 × 10^−3^ mm^2^/s). Diffusion was measured at values of b = 0 s/mm^2^ and b = 400 s/mm^2^, according to the meta-analysis that suggested low-b-value as more valuable parameters than standard-b-value DWI for discriminating malignant from benign vertebral compression fractures.^20^

Considerable technical variability of the DWI exists among different institutions (such as Echo Planar Imaging or Fast Spin Echo methods, fat suppression methods, and selected b-values).^20,21^ Owing to the lack of standardization in DWI protocols and technical factors, quantitative measurements derived from DWI may have limited reproducibility with frequent substantial overlap between the cut-off values, reducing their applicability in clinical practice.^22^

An interpretation of ADC values is complex and varies significantly depending on the histology characteristics of the lesions. Although the ADC values of benign VBMLs are higher than malignant ones^8,9^, certain benign lesions, such as hyperplastic bone marrow have low ADC values due to the preserved bone and bone marrow structures.^23^ Additionally, the usefulness of ADC values in differentiating malignancy from infection was not consistently demonstrated across the studies.^22,24^ Furthermore, according to a study by Maeda *et al*., the false negative results of a malignant vertebral compression fracture may appear due to the necrotic tumor tissue, a large amount of associated interstitial edema, and an increased perfusion fraction in the hypervascular portion of the lesion.^25^ Summarized data of DWI characteristics in different studies are given in Table 2.

**TABLE 2.** The studies of diffusion-weighted imaging in the differentiation of bone marrow lesions

**Authors**	**No. of lesions**	**Clinical features**	**Technical parameters No. of image planes**	**Technical parameters b values (s/mm^2^)**	**ADC cut-off values (× 10^−3^ mm^2^/s)**
Park *et al*.^9^	86	Traumatic CFs *vs*. tumor infiltration with/without malignant CFs	Single shot SE EPI	0, 400, 1000	1.14
Kwack *et al.*^10^	126	Focal benign lesion *vs*. metastases	Single-shot echo-planar	0, 800	0.995
Geith *et al*.^12^	46	Osteoporotic *vs*. malignant CFs	Single shot TSE	100, 250, 400	1.7
Park *et al*.^23^	58	Hyperplastic hematopoietic BM *vs*. malignant BM lesions	Single shot SE EPI	0, 800	0.695
Schmeel *et al*.^27^	89	Benign (traumatic, inflammatory, and primary) *vs*. malignant (metastatic and hematologic)	Single-shot spin-echo echo-planar with multislice short TI inversion recovery fat suppression	0, 800	1.08
Pozzi *et al*.^29^	116	Benign primary tumors *vs*. bone metastases *vs*. malignant primary tumors	Spin-echo echo-planar technique	0, 1000	0.952 (benign *vs*. malignant tumors)
Hajalioghli *et al*.^30^	23	Atypical hemangiomas and metastases	Spin-echo single-shot echo-planar with fat suppression	50, 400	0.958
Lee *et al*.^31^	51	Schmorl nodes *vs*. bone metastases	Single-shot (FOCUS, GE Healthcare)	0, 400, 1000	1.028

ADC = apparent diffusion coefficient; BM = bone marrow; CF = compression fracture; DWI = diffusion-weighted imaging, EPI = echo planar imaging; SE = spin echo; TSE = turbo spin echo

The cut-off ADC values obtained in our study were comparable to the results of Park HJ *et al*. and Geith *et al*.^9,12^ The similarity of technical conditions and prevalence of benign fractures among benign lesions across all studies could be a reason for concordant results. On the contrary, Kwack *et al*. reported significantly different ADC cut-off values compared to our results (≤ 995 × 10^−6^ mm^2^/s versus ≤ 1.57 × 10^−3^ mm^2^/s).^10^ The authors compared benign VBMLs and metastases, but the benign compression fractures and Schmorl's nodes were excluded from the analysis. Suh *et al*. reported a sensitivity of 89% and specificity of 87% of ADC for differentiating benign and malignant VBMLs and compression fractures, similar to our results.^26^

In our study, the diagnostic accuracy was additionally improved with the combination of CSI IP-OOP and DWI techniques, with a sensitivity of 100% and specificity of 90.9% compared to either single quantitative assessment, with a cut-off value of > 0.19. Combined CSI IP-OOP and DWI techniques had an AUC of 0.988 (95% CI, 0.872–1.000). Similar improvement in the diagnostic accuracy with a combination of CSI and DWI was reported by Schmeel *et al*. in the analysis of benign VBMLa (traumatic, inflammatory, and primary spine tumors) versus malignant (metastatic and hematologic).^27^

Diagnosis of multiple vertebral lesions with similar MRI morphologic appearances is typically not in question and is usually attributed to metastases. Conversely, identifying the etiology of solitary lesions presents a greater challenge. Our findings demonstrate that chemical-shift and diffusion-weighted MRI can detect subtle differences between malignant lesions and their surrounding microenvironments compared to benign lesions. Therefore, these two non-standard MRI techniques might be effectively applied to clarify the diagnosis of solitary vertebral lesions using quantitative parameters, such as SIR and DWI.

Our study had several limitations. First, the study group demonstrated histological diversity among VBMLs. Although metastases were the most prevalent malignant type, variations within both benign and malignant histological types were present. Additionally, the sample size for the benign group was small. Future studies with larger sample sizes are warranted to overcome this limitation and ensure a more accurate differentiation of VBMLs. Second, not all VBMLs were histopathologically proven. Third, the subjectivity of readers during ROI selection could alter the values of SIR and ADC. Fourth, age and hematopoietic status can influence vertebral marrow composition, and are in correlation with both methods, CSI and DWI.^28^ Although the majority of our patients were female patients after natural or artificial menopause, we did not include hormonal and hematological data in our study.

In conclusion, the non-standard MRI techniques, CSI IP-OOP, and DWI, can significantly enhance the diagnostic accuracy of MRI in distinguishing between benign and malignant VBMLs. Moreover, the synthesis of CSI IP-OOP and DWI can further augment the diagnostic precision of MRI spine examinations. The capacity of non-standard MRI techniques to detect subtle, histologically diverse pathological processes within vertebral body marrow emphasizes the imperative need for standardization of MRI techniques, and the analysis of larger and more homogeneous histological lesion types. This will undoubtedly contribute to the broader application of non-standard MRI techniques in routine clinical assessments of VBMLs.
